# Electrospinning of Nanofibrous Membrane and Its Applications in Air Filtration: A Review

**DOI:** 10.3390/nano11061501

**Published:** 2021-06-06

**Authors:** Chenxin Lyu, Peng Zhao, Jun Xie, Shuyuan Dong, Jiawei Liu, Chengchen Rao, Jianzhong Fu

**Affiliations:** 1 The State Key Laboratory of Fluid Power and Mechatronic Systems, Zhejiang University, Hangzhou 310027, China; lyuchenxin@zju.edu.cn (C.L.); jxie93@zju.edu.cn (J.X.); 21925168@zju.edu.cn (J.L.); raochengchen@bytedance.com (C.R.); fjz@zju.edu.cn (J.F.); 2 Key Lab of 3D Printing Process and Equipment of Zhejiang Province, Zhejiang University, Hangzhou 310027, China; 3 School of Mathematics, Jilin University, Changchun 130012, China; dongsy1019@mails.jlu.edu.cn

**Keywords:** electrospinning, nanofibrous membrane, air filtration, particulate matter

## Abstract

Air pollution caused by particulate matter and toxic gases is violating individual’s health and safety. Nanofibrous membrane, being a reliable filter medium for particulate matter, has been extensively studied and applied in the field of air purification. Among the different fabrication approaches of nanofibrous membrane, electrospinning is considered as the most favorable and effective due to its advantages of controllable process, high production efficiency, and low cost. The electrospun membranes, made of different materials and unique structures, exhibit good PM2.5 filtration performance and multi-functions, and are used as masks and filters against PM2.5. This review presents a brief overview of electrospinning techniques, different structures of electrospun nanofibrous membranes, unique characteristics and functions of the fabricated membranes, and summarization of the outdoor and indoor applications in PM filtration.

## 1. Introduction

Air pollution caused by ambient pollutants such as particulate matter (PM2.5, PM10, and bacteria) and toxic gases has always been a shared problem faced by many countries in the world. The PM2.5, being the most common air pollutant, is posing a tremendous threat on the global public health and economy. The infamous PM2.5 is notorious for its hazardous nature of penetrating into the respiratory system of human, and delivering harmful chemical compositions through blood system, thus chronically damaging human respiratory and cardiovascular systems [1,2]. An increasing amount of evidence has indicated that both long-term and short-term exposure to ambient PM2.5 air pollution is closely related to cardiovascular, respiratory, and cerebrovascular diseases, [3] even cognitive impairment [4]. Besides, exposure to PM2.5 can result in an economic loss in typical cities, with individuals suffering from financial damages of welfare loss [5]. Therefore, it is highly desirable to apply air purification technologies on the alleviation of the harm caused by PM2.5 pollution for protection of individual health.

**Figure 1:**
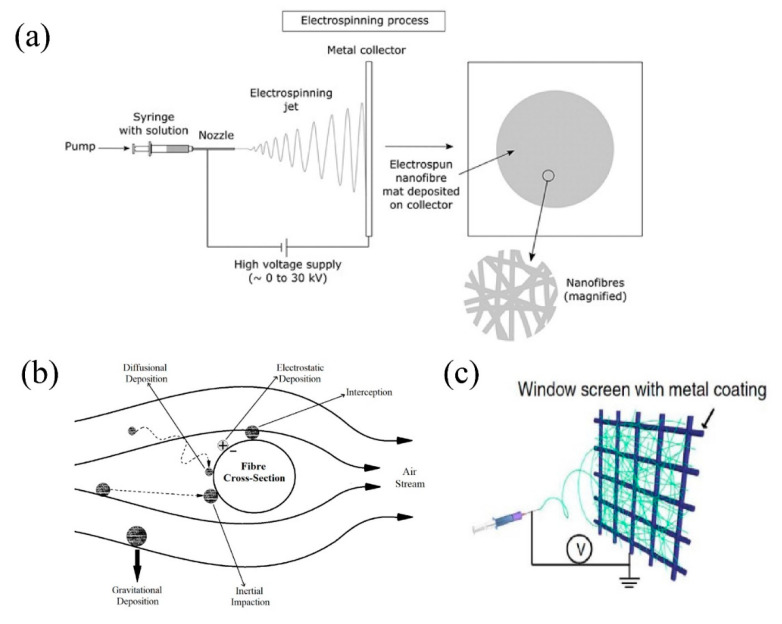
(**a**) Schematic diagram of processing steps for the fabrication of nanofibers. Reprinted with permission from ref. [15]. Copyright 2017 Taylor & Francis. (**b**) Main filtration mechanism of air filter. Adapted with permission from ref. [6]. Copyright 2020 Elsevier B.V. (**c**) Schematics showing the fabrication of transparent air filter by electrospinning. Adapted with permission from ref. [16]. Copyright 2015 Macmillan Publishers Limited.

As challenging as it is, there are several effective methods to tackle air pollution problem. Multiple air cleaning methods are basically categorized into ionizers, ozonizer, electrostatic precipitator, photo-catalytic oxidation, adsorption, and solid media filtration [6]. Among these methods, solid media filtration or membrane filtration, using porous, woven or non-woven membrane as filter medium, is highly capable of filtering PM2.5 off the air. Porous air filter capturing PM particles is illustrated in Figure 1b. To evaluate the filtration performance of membranes, the quality factor (QF) is introduced, which indicated that a filtration membrane with a high QF should satisfy two qualifications: high filtration efficiency and low pressure drop [7].

For the material of air filtration membrane, nanofiber has gained increasing interest from researchers due to its wide applications in various disciplines such as filter medium, tissue engineering scaffolds, composite materials, artificial blood vessels, biochips, drug delivery, nanosensors, optics, etc., [8,9,10]. Moreover, its unique advantages of large specific surface area and ultra-fine porosity make it a promising candidate for air filtration membrane material. Nanofiber’s major special feature is the large surface area, which leads to the increase of surface energy and activity, resulting in the surface and interface effects, the quantum tunneling effect, the small size effect, and the quantum size effect. Because of these effects, the chemical and physical properties of nanofiber are changed. There are many kinds of existing methods for preparing nanofibers, including electrospinning technology, stretching method, self-assembly, template synthesis, and microphase separation. Among them, the electrospinning method utilizes high-voltage static electricity to form a jet of polymer melt or solution. The jet travels straightly in the beginning and helically with the gradual increase of the travel distance, and accompanied by the volatilization of the solvent, the nanofiber filaments are finally deposited on the surface of the receiving device to form a nanofiber filter membrane, and the nanofiber filter membrane has good air permeability, warmth retention and barrier properties [10]. Characteristics of electrospun nanofibrous membranes, such as low Knudsen number, [11] slip flow effect causing the decrease of pressure drop, [12] and the formation of garland leading to better filtration efficiency, [13] etc., make the nanofibrous membranes ideal filtration media. The electrospinning method has the advantages of simple manufacturing equipment, wide application range, controllable process, high production efficiency, and low cost. It can spin fibers of different shapes and orientations as required to meet various performance requirements, and is considered as one of the most effective means for nanofiber preparation [14].

**Figure 2:**
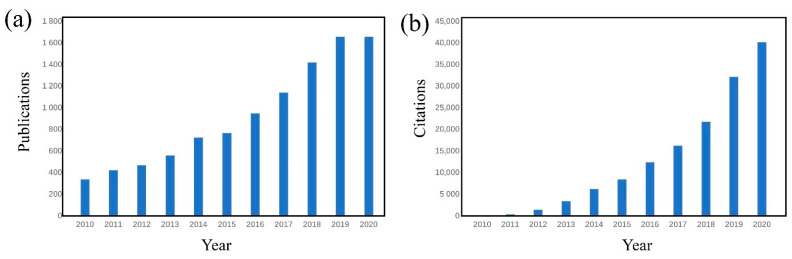
The statistics data of the publications on the topic electrospinning membranes from Web of Science. (**a**) Publications and (**b**) citations in each year. Data were collected in the past 10 years (2010–2020).

In recent years, the number of publications on the topic of electrospinning membranes has increased; in 2020 publications on this topic even reached 1653 (Figure 2), and the number of total patents reached over 120. As a well-explored research field of nanotechnology, there are already many reviews covering electrospinning techniques and various applications of nanofibrous membranes, including reviews by Robert et al. [6], Li et al. [7], Rasouli et al. [17], Barhoum et al. [18], and Zhu et al. [19]. Because of the swift development of electrospinning techniques, the research topic of electrospun membranes as air filtration medium is gaining focus. Despite these previous works, our review, covering mainly the latest progress and contributions, servers as a renewal for this topic, provides summarization and comparison of novel electrospinning techniques, electrospinning products with their new applications, and our views on future trends and perspectives. In this review, we provide an overview of electrospinning techniques, along with structures of electrospun air filtration membranes, then, functions and characteristics of filtration membranes are summarized, and finally, we focus on some applications of electrospun nanofiber air filtration membranes.

## 2. Electrospinning Techniques

Electrospinning machines mainly consist of high-voltage DC power supplies, injection pumps, spinning nozzles, and collecting devices. The process of fabricating nanofibers is: a metal wire connects the needle to the positive electrode of the high-voltage generator. The collecting device is a metal collecting plate disposed at the opposite end of the capillary. It may also be a metal surface or other collecting device such as a rotary drum to meet the conditions of different experiments. The collector plate is grounded with a wire as the negative electrode is connected to the high voltage power supply. The high-voltage electrostatic field generates a potential difference instantaneously between the capillary spinneret and the grounding electrode. Under a powerful electric field force, the polymer solution or melt (generally non-Newtonian fluid) overcomes its surface tension and viscoelastic force to generate charge, the mutual exclusion between charges, and the opposite charge electrode’s compression to the surface charge, directly creating a force that is opposite to the surface tension. When the electric field strength exceeds a certain critical value, the polymer solution or melt will overcome the surface tension of the droplet and form a jet. After being stretched into a straight line to a certain distance, it is sprayed along a spiral path. A semi-spherical cone of droplets appears at the end of the spinneret, which is called a Taylor cone, and as the solvent volatilizes, cools, and solidifies, it finally deposits on the collection device to form fibers. The electrospinning process of air filter membranes is shown in Figure 1a.

Divided by the state of the polymer used to fabricate nanofiber membranes, electrospinning can be categorized into solution electrospinning and melt electrospinning [20], the latter is also known as solvent-free electrospinning [21]. The most conventional and most widely used solution electrospinning, or single nozzle electrospinning, is relatively simple to set up and operate, but the low production rate and residue of organic/toxic solvent in nanofibers remain as the major challenges. The leading research progress of needleless electrospinning and multi-needle electrospinning shows a trend of fast and up-scaling production of electrospinning membranes, which solidifies the foundation of their expanded applications in air filtration. Melt electrospinning reduces the usage of toxic solvents, thus it is considered as more environmentally friendly than solvent electrospinning.

### 2.1. Needleless Electrospinning

**Figure 3:**
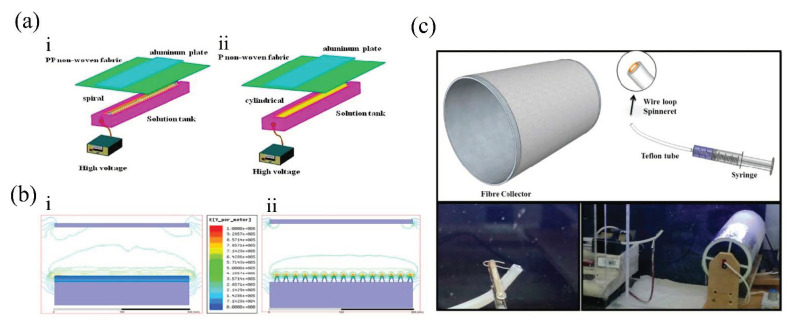
(**a**) Schematic diagrams of the homemade electrospinning setup: i. spiral electrode and ii. cylindrical electrode. Reprinted with permission from ref. [23]. Copyright 2019 John Wiley & Sons, Ltd. (**b**) Electric field profiles of: (**i**) spiral electrode, (**ii**) cylindrical electrode. Reprinted with permission from ref. [23]. Copyright 2019 John Wiley & Sons, Ltd. (**c**) Schematics and photo of the experimental setup of wire loop spinneret. Reprinted with permission from ref. [25]. Copyright 2018 Wiley-VCH Verlag GmbH & Co. KGaA, Weinheim.

Due to the absence of needle-like spinnerets, needleless electrospinning is immune to the common problem of clogging and low productivity faced by single needle electrospinning. Unlike the needle and syringe used in single nozzle electrospinning, the needleless electrospinning system often contains a large-surfaced, rotating or stationary spinneret to guarantee that the electric field is strong enough to allow single or multiple polymeric jets eject from the surface of the polymer solutions. For example, Ng et al. [22] developed a rotating-disk electrospinning system (Figure 3a) to fabricate poly(caprolactone)(PCL), poly(lactic acid)(PLA), and poly(vinyl alcohol)(PVA) nanofiber mats, and compared them with the conventional single nozzle electrospinning. This needleless electrospinning method exhibited a much higher productivity than the single nozzle electrospinning and was proved to be more easily operated, with no clogging existing, and capable of forming nanofiber mats at relatively low voltages. Besides, rotating-disk electrospinning could be used with different polymers of different solvent systems. Like the rotating-disk mentioned above, electrodes often came in different shapes, which had different effects on the quality of the fabricated membranes. Yan et al. [23] reported the fabrication of ultrafine polyamide 6 (PA-6) nanofiber membranes via needleless electrospinning process, during which the relative humidity condition and electrode type were both controlled. The cylindrical electrode and the spiral electrode were used separately to form PA-6 nanofiber membranes (Figure 3b). The results showed that the membrane prepared by the spiral electrode was finer than that by the cylindrical electrode, because of the tip effect and higher electric field intensity of the spiral electrode (Figure 3c). The PA-6 membrane formed by the spiral electrode exhibited high PM filtration efficiency of 99.42% and low pressure drop of 85.5 Pa. Moon et al. [24] developed a helically probed rotating cylinder (HPRC) system based on needleless electrospinning and chemical vapor deposition (CVD) to form the polyacrylonitrile (PAN) nanofiber membrane. The HPRC system could fabricate a large amount of PAN nanofiber in an hour with uniform morphology, profoundly raising the production rate of electrospun nanofiber membranes. Jahan et al. [25] reported a novel spinneret from a tube with a single wire loop embedded in its one end with a controlled feeding of solution. Compared with needle electrospinning, this wire loop spinneret generates a stronger electric field, and the production rate of nanofiber membranes was 0.48 $g\cdot h^{-1}$. Zhou et al. [26] designed a stepped pyramid spinneret to fabricate three-dimensional polyacrylonitrile/polyimide (PAN/PI) composite nanofiber membranes, and the yield was 120 times that of single needle electrospinning. According to the advanced research mentioned above, it is apparent that needleless electrospinning is a promising way to lift the production rate of nanofiber membranes, compared with its needle electrospinning rival. Different designs of needleless electrospinning spinneret are summarized in Table 1.

### 2.2. Multi-Needle Electrospinning

**Figure 4:**
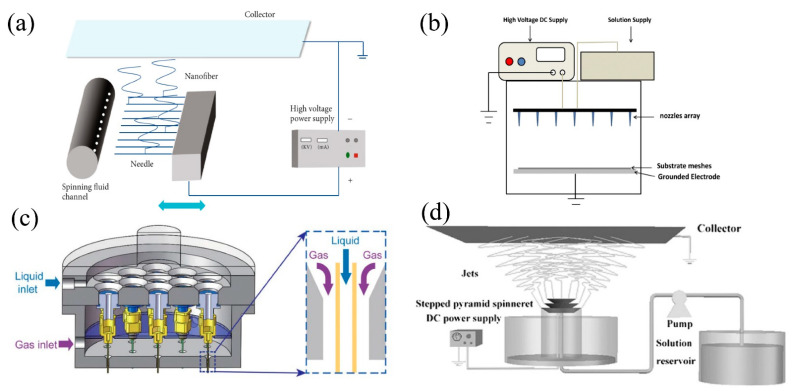
(**a**) Schematic diagram of the multi-needle external liquid e-spinning device. Reprinted with permission from ref. [35]. Copyright 2020 Yuan Xu et al. (**b**) Schematic diagrams of the needle-based electrospinning machine. Reprinted with permission from ref. [37]. Copyright 2020 Rongguang Zhang et al. (**c**) Structure of the sheath gas constrained multinozzle spinneret. Reprinted with permission from ref. [39]. Copyright 2019 Wiley Periodicals, Inc. (**d**) Scheme of the electrospinning apparatus using a stepped pyramid spinneret. Reprinted with permission from ref. [40]. Copyright 2017 Wiley-VCH Verlag GmbH & Co.KGaA, Weinheim.

Due to the use of low viscosity polymer solutions and evaporation during the needleless electrospinning process, the production rate is not high enough [27]. In the multi-needle electrospinning, with the increase of the number of nozzles, the production rate is greatly raised [15]. The mechanism of multi-needle electrospinning is similar to the conventional single needle electrospinning, and like needleless electrospinning, multi-needle electrospinning system usually includes a specially designed spinneret with a unique structure, which contains several nozzles working simultaneously. Researchers have designed different-shaped spinnerets and successfully applied them in electrospinning process. Xu et al. [35] proposed a multi-needle electrospinning device for large-scale production of thermoplastic polyurethane (TPU) nanofiber membranes. The spinneret was cylindrical, with multi-needle linearly aligned on the surface, and was filled with polymer solution (Figure 4a). The sliding table continuously moved left and right to draw the solution, and under a high voltage, a plug fluid jet was formed and solidified into a collector deposited on the fiber. The linear arrangement of needles was proved effective to improve the yield because each needle surface could form multiple jets at the same time. Jiang et al. [36] developed a one-step 12-needle electrospinning method with liquid bath circulating system to form PA6/CS-NPs nanofiber membranes. Zhang et al. [37] developed a multi-needle electrospinning equipment and successfully fabricated nanofiber filters with antibacterial and deodorizing functions. The multi-needle electrospinning equipment consisted of a syringe pump with an array of nozzles, and the fabricated membranes exhibited great filtration performance of PM (Figure 4b). To tackle the problem of nonuniform electric field of the needle tip caused by the overly intensive arrangement of needles, Zhu et al. [38] studied the influence of the needle size and the dielectric material on the electric field of the tip. The results showed that using dielectric material on the tip of the middle part of the needle contributed to the electric field uniformity in case of high-density arrangement of the needle, and the electric field intensity was increased by 1.21 times. To overcome the problem of mutual interferences among the nozzles, Zheng et al. [39] designed a multi-needle spinneret with sheath gas as an additional stretching force, and to boost the simultaneous ejection of multiple jets (Figure 4c). With the introduction of sheath gas, the resultant productivity reached 0.618–0.712 $g\cdot h^{-1}$, which was 30–50 times as that of the conventional electrospinning. Multi-needle electrospinning exhibits even better production rate than the needleless electrospinning, making the scale-up of nanofiber membranes fabrication possible.

### 2.3. Solvent-Free Electrospinning

**Figure 5:**
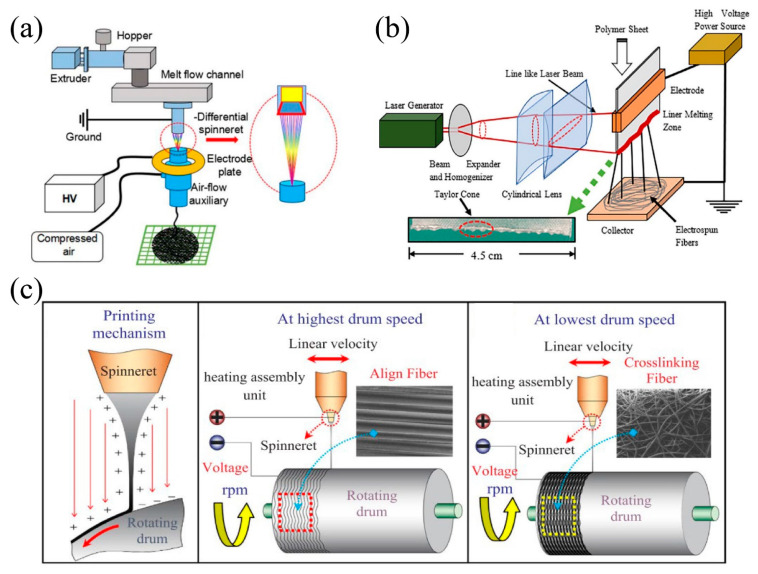
(**a**) Schematic diagram of melt differential electrospinning device. Reprinted with permission from ref. [42]. Copyright 2018 Wiley Periodicals, Inc. (**b**) Schematic illustration of a line-like CO2 laser M-ESP device. Reprinted with permission from ref. [43]. Copyright 2019 Society of Plastics Engineers. (**c**) Composite PEBA fiber synthesis mechanism for Melt-electrospinning process. Reprinted with permission from ref. [46]. Copyright 2019 Elsevier Ltd.

Other eye-catching electrospinning method is solvent-free electrospinning, or melt electrospinning. Without toxic solvent, the solvent-free electrospinning can achieve environment-friendly fabrication of nanofiber membranes. Polymer is directly heated for the formation of fibers in melt electrospinning and the produced fibers have fewer defects and better mechanical properties besides higher yield [41]. As for the research progress of melt electrospinning, Qin et al. [42] successfully fabricated PLA nanofibers with the diameter of 236 mm by melt differential electrospinning. A special designed differential spinneret was used, and nontoxic acetyl tributyl citrate (ATBC) was added by airflow (Figure 5a). The results indicated an accelerated falling speed of the jets of 347 times of that without airflow, and proved that the combination of ATBC and airflow was a good strategy for mass production of nanofibers with excellent stability, being both efficient and eco-friendly. Zakaria et al. [43] developed a melt electrospinning technique using a ${\mathrm{CO}}_{2}$ laser melting device to fabricate delicate polypropylene (PP) nanofibers with added polyvinyl butyral (PVB). The melt electrospinning system with a line-like laser melting device formed many Taylor cones simultaneously, and every single Taylor cone was an individual fiber production unit, therefore a high production rate was guaranteed (Figure 5b). Buivydiene et al. [44] designed a novel fiber-printing apparatus following the principles of additive printing and melt electrospinning to produce fiber mats. During the electrospinning process, parameters such as tip-to-collector distance and temperature could be controlled. Five polyamide and polyolefin-based polymers were tested, and a wide range of fiber morphologies and high dispersion of fiber diameter were observed. Sarwar et al. [45] produced poly(ether-block-amide)(PEBA) fibers by melt electrospinning, and studied the effect of parameters on fiber diameter. The results showed that with the increasing of voltage, distance, collector speed and melting temperature, the fiber diameter decreased, and when these parameters went above a certain limit, the fiber diameter was further increased. Sarwar et al. [46] also developed a new approach to produce nanocomposite PEBA filaments with average diameter of 1.6 mm with uniform nanofiller dispersion which could be used in melt electrospinning. The designed melt electrospinning device included a cylindrical rotating drum as the receiver (Figure 5c). At the highest and lowest drum speed, align fiber and crosslinking fiber were formed respectively. Li et al. [47] constructed a nanofiber membrane with a hierarchical fibrous structure using a combination of PP melt-blown and PVA/ZIF-8 electrospinning technique. The fabricated membrane exhibited excellent filtration efficiency of 96.5% for PM2.5, tensile strength of 33.34 N, and low pressure drop.

### 2.4. Discussion

Compared with the most common single needle electrospinning, other electrospinning techniques mentioned in this chapter, including needleless, multi-needle, and solvent-free electrospinning, have unique merits suitable for different fabrication demands, and drawbacks that need to be considered. High production rate is the most basic requirement for the large-scale production and wide-spread applications of nanofibrous membranes, needleless and multi-needle electrospinning are promising techniques because the electrodes and spinnerets of novel structures, which are significant for the simultaneous formation of multiple polymer jets, are applied in both needleless and multi-needle electrospinning. However, clogging is still a disadvantage of multi-needle electrospinning. When the clogging of specific nozzle happens, the electrospinning liquid is redirected to other working nozzles, which deoptimizes the spinning of the specified nozzle. The needleless electrospinning technique abandons needles and generates polymer jets with various spinneret design such as wire/coil, disk, plate, and sprocket wheel. Compared with the multi-needle electrospinning, the common problem of clogging is terminated and a more stable production is therefore guaranteed. The relatively easy maintenance of needleless electrospinning set-up is another advantage, which can save the time and finance cost in the production process. Multi-needle electrospinning, containing several nozzles, is capable of finer control of the fiber distribution. Unlike needleless electrospinning, there is less evaporation during the multi-needle electrospinning process, which results in the higher production rate than needleless electrospinning. Environment-friendless is another demand of the large-scale production of nanofibers, and melt electrospinning is promising because of the lack of toxic solution in the electrospinning process. Similar to the needleless electrospinning, without the application of nozzles, the control difficulty still exists in melt electrospinning. Brief summarization and comparison of different electrospinning techniques are listed in Table 2.

## 3. Structures of Membranes

Structures of electrospun membranes is an important factor that greatly influences the filtration performance and functions of the air filter. With electrospinning techniques, different, versatile and unique structures are possible to be designed and fabricated, leaving great room for improvement in the quality factor of the nanofiber membrane. Classical structures such as bead-on-string and multilayer have been widely and deeply studied to optimize the air filtration performance and to endow multiple functions to nanofiber membranes.

### 3.1. Bead-on-String Structure

The nanofibrous membrane with bead-on-string structure usually exhibits a relatively high filtration efficiency and a low pressure drop. The bead-on-ring structure increases the distance between nanofibers and reduces the volume fraction of the membrane, allowing air flow through the filter [48]. This structure is shaped by a few parameters of the electrospinning process. Korycka et al. [49] carried out a series of experiments to study the influence of several factors such as the flow rate, viscosity of polymer solution, and applied electrical voltage, etc., on the average diameter of fibers and beads produced by electrospinning process. The greatest influence on the diameter of beaded fibers and beads was observed for the dynamic viscosity of the feed solution, and the electrical voltage was the second major factor contributing to the size of the fiber and bead. Rasouli et al. [50] studied the evolution of the bead-on-string morphology of electrospun polysolfone (PSU) mats and correlated it with solution concentration, voltage, and feed rate. The results revealed that the bead on string structure was formed at concentrations between 7 and 18 wt% PSU, and the number of beads decreased as either the feed rate or the voltage increased. Besides, influenced by the increasement of voltage, which raised the aspect ratio of the beads, the morphology of the beads transformed from spherical to spindle-like.

**Figure 6:**
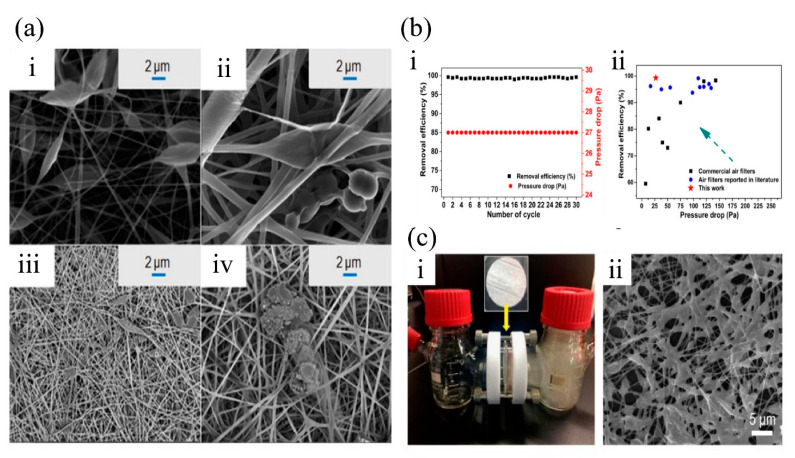
(**a**) Effect of electrospinning distance on the morphology of PLA-PSQ fibrous membranes ((**i**–**iv**) represent the receiving distances of 8, 12, 16, and 20 cm, respectively). Reprinted with permission from ref. [51]. Copyright 2019 The Polymer Society. (**b**) (**i**) Filtration efficiencies of a bead-on-string filter in 30 cycles; (**ii**) comparison figure of the bead-on-string filter with other filters reported in literatures and commercial filter. Reprinted with permission from ref. [52]. Copyright 2019 Elsevier B.V. (**c**) (**i**) Demonstration of GOPAN filter to shut off PM from the outdoor (right bottle). (**ii**) SEM image of GOPAN membrane after filtration test. Reprinted with permission from ref. [53]. Copyright 2018 Elsevier B.V.

The bead-on-string structure significantly improves the air filtration performance. Cao et al. [51] studied the optimum conditions for the bead on string fibrous membrane based on polysilsesquioxane (PSQ)-immobilizing poly (lactic acid) (PLA-PSQ). The PLA-PSQ membrane exhibited higher air filtration efficiency and lower pressure drop, because the results showed that the fiber formed evenly distributed beads and larger pore sizes more easily and the beads increased the distances between fibers, which lowered the pressure drop (Figure 6a). Besides, the immobilization of PSQ to PLA decreased the loss of PSQ, causing better hydrolytic resistance. Huang et al. [52] fabricated bead on string filters with nanobeads along the nanofiber axis by optimizing the PAN concentration and ambient humidity condition during the electrospinning process. The two parameters of polymer concentration and humidity condition together shaped the desirable bead on string morphology (Figure 6b). The PAN filter reached the filtration efficiency of above 99%, which was achieved by the ultrafine nanofibers, and the pressure drop was 27 Pa, as the result of nanobeads reducing the packing density. Li et al. [53] fabricated GOPAN composite nanofibrous membranes with an olive-like bead-on-string structure, which exhibited filtration efficiency of 99.97% with pressure drop of 8 Pa (Figure 6c). Their research showed that the unique olive-like bead structure contributed greatly to the air filtration performance by increasing the interfiber distance and specific surface area and surface functional groups. The above research progress proves that the bead-on-string electrospun membranes are capable of filtering PM2.5 off the air with a relatively high filtration efficiency and low pressure drop, which meets the basic need of the air filters.

### 3.2. Multilayer Structure

**Figure 7:**
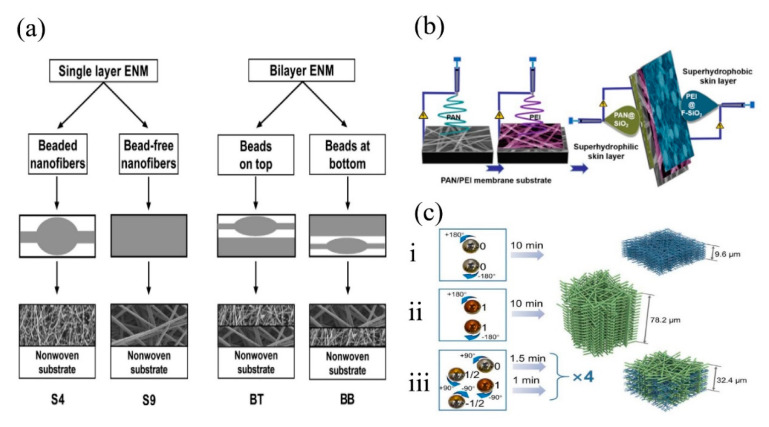
(**a**) Schematic representation of single and bilayer ENMs in the experimental design where S represents a single layer and B is bilayer ENM. Reprinted with permission from ref. [54]. Copyright 2019 Elsevier B.V. (**b**) Schematic illustration of the fabrication of the PAN/PEI bilayer membranes and the asymmetric superwettability skin layers. Reprinted with permission from ref. [56]. Copyright 2020 Yuyan Yang et al. (**c**) The fabrication of: (**i**). nanofibers assembled membrane (PAN-10); (**ii**). submicron fibers assembled membrane (PAN-16); (**ⅲ**). submicro-/nanofibers sandwich-structured membrane (PAN-10/16). Reprinted with permission from ref. [59]. Copyright 2018 IOP Publishing Ltd.

As its name suggests, multilayer structured membranes are usually made up of two, three, or more layers of nanofibers. The structure and material of each single layer often vary, and layers of different structures are stacked in a specific order. Kadam et al. [54] studied an advanced combination of bead and bead-free bilayer electrospun nanofiber membranes to capture PMs. Two different bilayer PAN membranes were prepared, one with the bead layer on top of the bead-free layer and the other in reverse (Figure 7a). The results indicated that the stacking order of two layers had a nonnegligible impact on air filtration performance. The membrane with bead-free layer being on top and bead layer at bottom reached the filtration efficiency of 95.7% and reduced the pressure drop to 137 Pa, superior to the membrane in the reversed stacking order with filtration efficiency of only 95% and pressure drop of 202 Pa. Besides, the fabricated bilayer-beaded membrane was capable of effectively filtering PM particles at a relatively small basis weight, making it an ideal choice for respirator mask and protective clothing. Other special features can be achieved with the combination of layers made of different materials. Zhang et al. [55] fabricated multilayer membranes with antibacterial ability via sequential electrospinning. The three-layer electrospun membrane was constructed with PVA/P(ADMH-NVF) as the middle layer, and PVA/CS on both sides. This multilayer membrane had relatively smooth fiber surface, excellent mechanical properties, and a small basis weight. Yang et al. [56] designed and fabricated a multilayer air filtration mask with a highly breathable and thermal comfort membrane combined with asymmetrically superwettable skin layer via electrospinning (Figure 7b). The membrane displayed a low basis weight of 3.0 $g\cdot m^{-2}$, a good air permeability of 278 $\mathrm{mm}\cdot s^{-1}$, a high filtration efficiency of 99.3%, and a low pressure drop of 64 Pa. Roche et al. [57] fabricated novel polyvinylidence fluoride (PVDF) nanofibrous multilayer membranes by wire-based industrial electrospinning equipment, and the results showed that the filtration efficiency of the membrane reached 99.00% for PM2.5. Wang et al. [58] developed a new multilayer membrane by incorporating ZIF-8 into polyacrylonitrile (PAN) to achieve high surface roughness, and the multilayer structure was formed by stacking of layers of rough microfibers with PAN layers via electrospinning. Due to its large specific surface area, rough fiber surface, and hierarchical pore structure, the fabricated membrane exhibited high filtration efficiency of 99.973% and pressure drop of 80.1 Pa. Xiong et al. [59] fabricated a low filtration resistance sandwich-structured PAN filters through a controlled accumulation of bimodal sized fibers (Figure 7c). The sandwich multilayer structure reduced the filtration resistance, and demonstrated a filtration efficiency of 99.89%.

### 3.3. Discussion

The bead-on-string and multilayer are two promising structures of electrospun nanofibrous filtration membranes because of their excellent filtration performance and antibacterial function, which are highly related to the fabrication process of these two structures. During the fabrication of the bead-on-string membranes, the structure is shaped by optimum control of parameters such as solvent concentration, ambient humidity, fiber distribution, etc., therefore the distance between nanofibers is increased and the volume fraction is reduced, finally leading to boosting filtration efficiency and decreasing pressure drop. The bead-on-string membranes are perfect for the application of air filters.

Compared with bead-on-string, multilayer structure is more complicated to fabricate. The multilayer membranes can tolerate multiple materials, different structures, and different stacking orders of each layer, which brings many combinations of materials and structures to the membrane, resulting in variation of filtration performance and realization of unique features. The specific stacking orders can be achieved by sequential electrospinning, and with the addition of other materials, unique characteristics such as antibacterial activity, good mechanical properties, small basis weight, high breathability, and good thermal comfort are made possible. Because of these characteristics, multilayer membranes outperform the bead-on-string ones in the application of wearable protections.

## 4. Characteristics of Membranes

Various functions and characteristics of electrospun membranes depend not only on structures, but also on the application of materials. In general, electrospinning materials should have a linear molecular structure. To form the membrane, the electrospinning material must be soluble, and its molecular weight must reach a certain amount.

Various polymers, including synthetic polymers and biopolymers, have been successfully applied to the electrospinning process, forming different nanofiber membranes. The most widely used synthetic polymers in electrospinning membranes are polyamide (PA) and polyacrylonitrile (PAN), and typical biopolymers include wool keratin, chitosan, polylactic acid (PLA), and bio-based PA-56 polymers [48].

Researchers have already developed high quality air filtration electrospun membranes, which possess high air filtration efficiency and low pressure drop. Instead of single material, these advanced air filtration membranes can be made of multiple kinds of polymers. The combinations of different polymers often result in the improvement of basic filtration performance measured by QF, and new features such as high thermal stability, antibacterial function are introduced. Following the concept of green electrospinning, which means using green materials, green solution, and green electrospinning method, [60] environment-friendly and biodegradable materials are gaining unprecedented popularity.

### 4.1. Enhanced Filtration Performance

**Figure 8:**
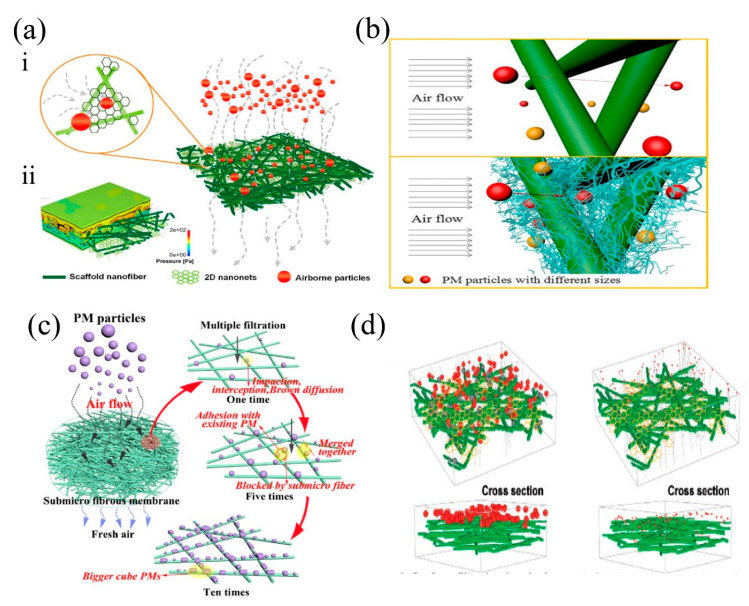
(**a**) 3D model illustrating the filtration process of PMIA NF/N membrane for 300–500 nm particles by physical sieving and surface filtration: (**i**) absolute removal manner and (**ii**) robust air permeability. Reprinted with permission from ref. [61]. Copyright 2017 Shichao Zhang et al. (**b**) Schematic illustration of the PM2.5 removal process. Reprinted with permission from ref. [65]. Copyright 2018 Elsevier B.V. (**c**) Schematic illustration of PMs captured by SMF membrane after different time sequences. Reprinted with permission from ref. [26]. Copyright 2020 Elsevier Inc. (**d**) Model illustrating the capture process of airborne particles by combing, sieving and adsorption capacity of PU nanofiber/nets filters. Reprinted with permission from ref. [63]. Copyright 2017 Wiley-VCH Verlag GmbH & Co. KGaA, Weinheim.

Since high filtration efficiency of PM and low pressure drop are the two most fundamental qualities of electrospun air filtration membranes, how to increase filtration performance is of top concern to researchers. Compared with commercial fibrous filters, electrospun membranes made of different nanofibers already possess a relatively high PM filtration efficiency. Conventional electrospun materials, including polyurethane (PU), polyacrylonitrile (PAN), polyvinyl chloride/PU, polyamide-56, poly(lactic acid), and nylon-66 have gained improved filtration performance [61]. Electrospun membranes made of single conventional polymer can display excellent filtration performance. For example, Liu et al. [16] studied different nanofibers and compared the effect of filtration, light transmittance, air flow with the capture rate, and lifetime through comparing five raw materials such as polyacrylonitrile (PAN), polyvinyl pyrrolidone (PVP), polystyrene (PS), polyvinyl alcohol (PVA), and polypropylene (PP), and found that nanofibers prepared by electrospinning could effectively improve the filtration efficiency of the filter membrane. The results showed that the PAN filter membrane with an average diameter of 200 nm works best. When the light transmittance is 77%, the filtration efficiency reaches 98.69%. Yun et al. [62] spun a polyacrylonitrile (PAN) nanofiber filter membrane with an average diameter of 270 to 400 nm. Compared with commercial filters, the pressure drop is greatly reduced and the filtration efficiency is improved. Zuo et al. [63] fabricated free-standing PU nanofiber/nets air filters, which could achieve filtration efficiency of >99.00% for PM1-0.5 and >99.73% for PM2.5-1, while maintaining high light transmittance of about 70% and low pressure drop of 28 Pa (Figure 8d). Zhang et al. [64] demonstrated a facile strategy to fabricate the ripple-like polyamide-6 nanofiber/nets (PA-6 NF/N) air filter via electrospinning process, and this PA-6 NF/N membrane reached a high filtration efficiency of 99.996% and robust QF of >0.11 ${\mathrm{Pa}}^{-1}$.

Although the above mentioned electrospun membranes made of single polymer show high filtration efficiency and low pressure drop, by adding other different agents into polymer, the filtration performance of electrospun membranes could be further improved. Zhong et al. [65] fabricated PMIA/${\;\mathrm{SiO}}_{2}$-NF fibrous membrane, including scaffold poly (m-phenylene isophthalamide) (PMIA) nanofibers, and intertwine ultra-fine ${\mathrm{SiO}}_{2}$ nanofilaments on its surface (Figure 8b). This membrane made of hybrid materials reaches the PM2.5 filtration efficiency of 97.33% and PM10 of 98.48%, far better than the bare PMIA membrane. Moreover, by introducing ${\mathrm{SiO}}_{2}$ into PET material, Guo et al. [66] found that ${\mathrm{SiO}}_{2}$ reduced the fiber diameter. The PET membrane was compounded into sandwich-structured composite needle felt (PET/${\;\mathrm{SiO}}_{2}$ NNF) by heat treatment, and the filtration results showed that PET/${\;\mathrm{SiO}}_{2}$ NNF had a lower rate of increase of pressure drop and a higher filtration rate. This research showed that ${\mathrm{SiO}}_{2}$ greatly improves the resistance growth and ash cleaning performance, and compared to commercially available PPS NF and PTFE CNF, this composite needle felt PET/${\;\mathrm{SiO}}_{2}$ NNF has longer service life by slowly increasing the resistance and stable ash cleaning performance for dust removal. Ruan et al. [67] designed electrospun synthesized polyacrylonitrile: ${\mathrm{TiO}}_{2}$ and polyacrylonitrile-co-polyacrylate: ${\mathrm{TiO}}_{2}$ composite nanofiber membranes by controlling the nanofiber diameter and membrane thickness and enable strong particulate matter adhesion to increase the absorptive performance. The filtration efficiency was close to 100% for all the tested particles for both PAN: ${\mathrm{TiO}}_{2}$ and PAN-co-PMA: ${\mathrm{TiO}}_{2}$, and they displayed excellent air permeability (284–339 mm/s). Yang et al. [56] fabricated a composite membrane of PAN/PEI, which exhibits a good air permeability of 278 mm $s^{-1}$, a high filtration efficiency of 99.3%, a low pressure drop of 64 Pa, and a quality factor of 0.1089 ${\mathrm{Pa}}^{-1}$. Li et al. [53] fabricated graphene oxide/polyacrylonitrile (GOPAN) composite nanofibrous membranes with an olive-like beads-on-a-string structure and with high porosity. The PM2.5 removal efficiency of GOPAN membrane exhibited the highest efficiency (99.97%) with a low pressure drop (8 Pa). The significant enhancement of the air filter properties is attributed to the GO and the designed olive-like bead macrostructures. Lee et al. [68] fabricated electrospun magnetic-luminescent Cu//Tb dual metal organic frameworks (MOFs)-incorporated side-by-side nanofibrous (SBS-NFs) membrane. The SBS-NFs membrane was composed of Cu-MOF/PAN at one side, improving the filtration efficiency and reducing the pressure drop, and Tb-MOF/PAN at another side, investigating the PM adsorption process through the changes in luminescence intensity. The Cu//Tb dual MOF-incorporated SBS-NFs membrane exhibited a high filtration efficiency (90.2%) and a reduced pressure drop (60.7 Pa). Zhou et al. [26] fabricated three-dimensional polyacrylonitrile/polyimide (PAN/PI) composite sub-micro fibrous membranes via free surface electrospinning, where the waste PI short fibers were utilized as raw materials (Figure 8c). The membrane showed superior filtration efficiency of 99.4% and low pressure drop of 124.6 Pa at normal face velocity of 5.3 cm $s^{-1}$. It is obvious that with the introduction of different agents into the polymers, the filtration performance of electrospun membranes is successfully boosted.

### 4.2. Thermal Stability

**Figure 9:**
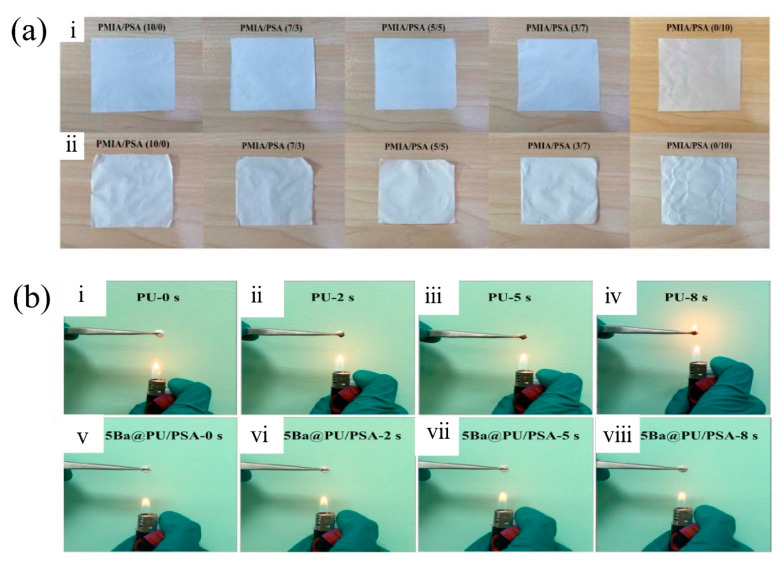
(**a**) Effect of heat treatment on PMIA, PSA and PMIA/PSA composite nanofibrous membranes, (**i**) before and (**ii**) after thermal exposure at 250 °C for 200 h. PMIA/PSA. Reprinted with permission from ref. [69]. Copyright 2019 IOP Publishing Ltd. (**b**) Combustion test of PU (**i**–**iv**) and Ba@PU/PSA (**v**–**ⅷ**) membrane. Reprinted with permission from ref. [70]. Copyright 2020 Elsevier B.V.

It is found that some agents, when composited with electrospinning polymers, could lead to special functions of membranes, thermal stability being one of them. This unique function increases the durability of the membrane at a relatively high temperature, making the electrospun air filtration membrane a promising choice of individual protection under extreme conditions. Tian et al. [69] proved that the filtration efficiency for PM2.5 of composite PMIA/PSA (7/3)(5/5) still remains as high as 99.9% even after being treated at 200 °C for 120 h, with PSA offering the strong thermal stability and thermal shrinkage performance and PMIA maintaining the high strength by providing good mechanical properties (Figure 9a). Similarly, Yang et al. [70] fabricated composite nanofibrous membrane through the blending spinning of PU and PSA and the introduction of ${\mathrm{BaTiO}}_{3}$, achieving high capture efficiency of 99.99%, low pressure drop of 39.4 Pa, good mechanical property of 13.27 MPa, high thermal stability up to 300 °C, favorable flame-retardancy and superior chemical resistance against acid and alkali (Figure 9b). Hao et al. [71] developed ZIF-8 modified soluble polyimide (PI) nanofibrous membranes via the electrospinning process. The prepared PI-ZIF membrane shows high PM 2.5 filtration efficiency of up to 96.6%, superior thermal stability of up to 300 °C, good mechanical properties, and low pressure drop.

### 4.3. Antibacterial Function

**Figure 10:**
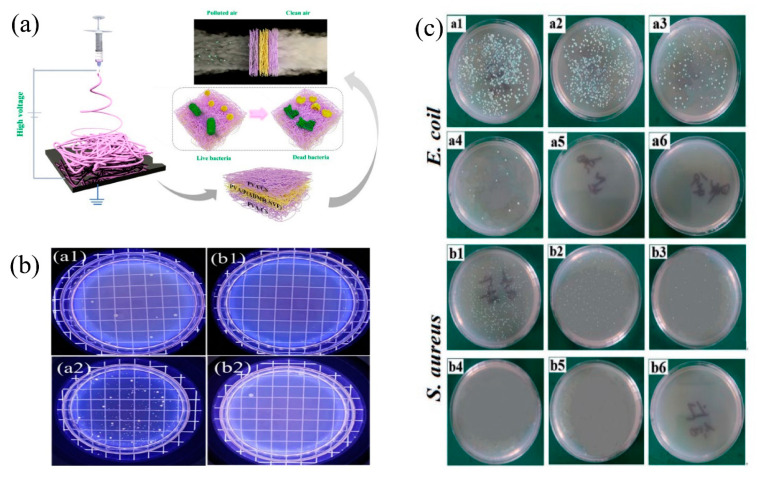
(**a**) Schematic illustration of fabrication of the multilayer membranes and application on the antibacterial air filtration. Reprinted with permission from ref. [55]. Copyright 2020 Elsevier B.V. (**b**) Representative images of the active colonies after 24 h incubation. Antibacterial efficacy of the samples (**a**) control, (**b**) PA6/CS-NPs against (**1**) *E. coli* and (**2**) *S. aureus*. Reprinted with permission from ref. [36]. Copyright 2020 Wiley Periodicals, Inc. (**c**) Photographs of surviving colonies of *E. coli* (**a**) and *S. aureus* (**b**) on nutrient agar dishes evaluated by the shake flask method for PAN/PU composite nanofibrous membranes with different added amount of ${\mathrm{AgTiO}}_{2}$ (**1**) 0.0% *w*/*w*, (**2**) 1.0% *w*/*w*, (**3**) 1.5% *w*/*w*, (**4**) 2.0% *w*/*w*, (**5**) 2.5% *w*/*w,* and (**6**) 3.0% *w*/*w*. Reprinted with permission from ref. [72]. Copyright 2018 Yanpeng Wu et al.

The polluted air often contains bacteria, and like PM2.5, these bacteria pose a threat to human health. The introduction of antibacterial agents into electrospinning materials gives the antibacterial function to the membranes, which protects individual’s health and safety. Researchers have developed electrospun membranes with antibacterial activity. Wu et al. [72] prepared polyacrylonitrile/polyurethane (PAN/PU) composite nanofibrous membranes with an antibacterial agent ${\mathrm{AgTiO}}_{2}$ via electrospinning method (Figure 10c). The filtration efficiency is 99.88% for particles of 0.3 mm, and >99.99% for airborne microorganisms with a 220.3 Pa pressure differential. This PAN/PU membrane with ${\mathrm{AgTiO}}_{2}$ was proved effective against both *Escherichia coli* and *Staphylococcus aureus.* Bortolassi et al. [73] fabricated and characterized Ag/PAN electrospun nanofibers, the Ag/PAN membranes exhibited excellent antibacterial activity against *E.coli* bacteria. Zhang et al. [55] synthesized a novel N-halamine biopolymer, P(ADMH-NVF) via free-radical copolymerization of N-Vinylformamide(NVF) and 3-allyl-5,5-dimethylhydantoin (ADMH), and combined with polyvinyl alcohol (PVA) as a middle layer (PVA/P(ADMH-NVF)). Polyvinyl alcohol/chitosan electrospun membranes (PVA/CS) were then orderly assembled onto both sides of the (PVA/P(ADMH-NVF)) membranes to form multilayer membranes. With the N-halamine structure, antibacterial activity was introduced, and the morphological changes of bacteria were observed (Figure 10a). Bortolassi et al. [74] developed a PAN nanofiber air filtration membrane with antibacterial function. They added three different particles of titanium dioxide (${\mathrm{TiO}}_{2}$), zinc oxide (ZnO), and silver (Ag) into PAN nanofibers, and the results showed that the ${\mathrm{TiO}}_{2}$ _F filter displayed the smallest fiber diameter and the highest filtration efficiency, and Ag_F filter showed a low pressure drop. Ag_F filter also showed good antibacterial function against *Escherichia coli.* These hybrid membranes proved that their quality factors were higher than commercially available nanofiber membrane for air filtration. Wu et al. [75] prepared an electrospun polyacrylonitrile/polyurethane (PAN/PU) composite nanofibrous membranes with an added antibacterial agent ${\mathrm{AgTiO}}_{2}$, and the filtration efficiency is 99.88% for 0.3 mm particles and >99.99% for airborne microorganisms with a 220.3 Pa pressure differential. Zhu et al. [76] fabricated a multifunctional poly (vinyl alcohol)/poly (acrylic acid) (PVA-PAA) composite membranes via green electrospinning and thermal crosslinking. Then superhydrophobic silica nanoparticles and ${\mathrm{AgNO}}_{3}$ were introduced, resulting in a rough surface and the formation of Ag nanoparticles through UV reduction. The fabricated membranes possessed high PM2.5 filtration efficiency of >98% and antibacterial and antiviral activities. With the combination of electrospinning polymers and other materials, antibacterial activity of electrospun membranes can be achieved while maintaining the good filtration performance.

### 4.4. Environment-Friendliness and Biodegradability

Green and biodegradable materials are gaining increasing popularity and focus among researchers due to its environment-friendly nature. The most common green and natural polymer materials, including cellulose, starch, chitosan, and proteinaceous materials such as wool and silk, are ideal polymer materials and easy to be decomposed by microorganisms, and green electrospinning materials produced from natural waste such as chitin, can become raw chemical materials after being processed [60].

**Figure 11:**
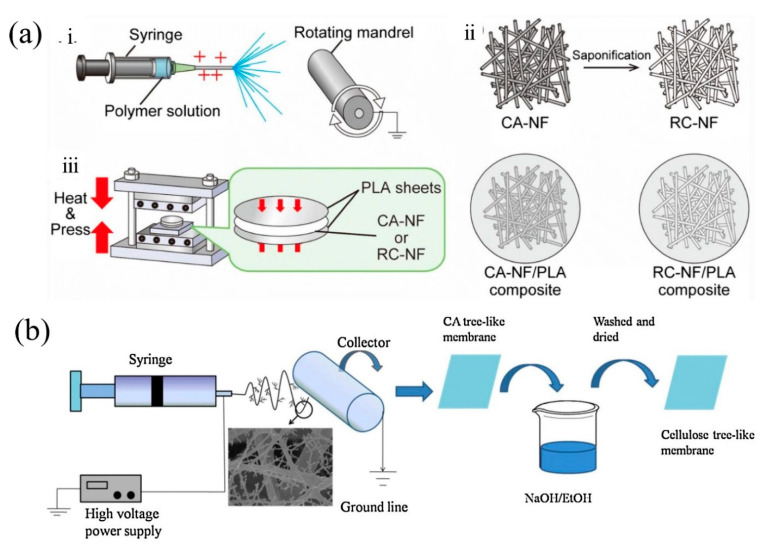
(**a**) (**i**) Electrospinning setup for fabrication of CA-NF; (**ii**) saponification of CA-NF to obtain RC-NF; (**ⅲ**) compression molding for the fabrication of CA-NF/PLA and RC-NF/PLA composite films. Reprinted with permission from ref. [78]. Copyright 2019 Springer Nature B.V. (**b**) Schematic diagram for the preparation of tree-like cellulose nanofibers. Reprinted with permission from ref. [79]. Copyright 2017 Elsevier Ltd.

As one of the most bountiful natural resources on earth, cellulose is well-known for its biocompatibility, biodegradability, physical strength, chemical resistance, thermal and mechanical properties, and regenerative and sustainable properties [77]. The application of cellulose in electrospinning is quite extensive. Kurokawa et al. [78] compounded cellulose-acetate nanofibers (CA-NF) and regenerated cellulose nanofibers (RC-NF) through electrospinning separately with polylactide (PLA), which is also a biodegradable material (Figure 11a). Because the mechanical property of PLA was not enough for industrial applications, CA-NF and RC-NF were introduced as reinforcement materials. Both CA-NF and RC-NF showed reinforcing efficiency for the improvement of the mechanical property of PLA. Zhang et al. [79] fabricated cellulose nanofiber membrane via electrospinning by adding certain amount of tetra butyl ammonium chloride (TBAC) into the cellulose acetate solution followed by a deacetylation treatment process (Figure 11b). The air filtration efficiency of cellulose acetate nanofiber membrane reached 99.58%. Ahne et al. [80] developed a cellulose-acetate-based nanofibers via electrospinning, and the maximum filtration efficiency measured was 99.8%.

Chitosan, or deacetylated chitin, is extensively applied in industry, agriculture, medicine, environmental protection, etc. As it is nontoxic and biodegradable, chitosan is a potential material for electrospinning to fabricate air filtration membranes. Zhu et al. [81] reported a multifunctional and bio-based chitosan/poly (vinyl alcohol) air filtration electrospun membrane. The superhydrophobic silica nanoparticles were introduced to increase filtration efficiency and the Ag nanoparticles were fabricated on the surface through UV reduction of ${\mathrm{AgNO}}_{3}$ to realize antibacterial function. The CS/PVA@${\mathrm{SiO}}_{2}$/Ag air filtration membrane possesses both excellent filtration performance and antibacterial activities, and has great potential application in individual protection against air pollution. Wang et al. [82] first prepared quaternary ammonium chitosan (HTCC) by chitosan and then electrospun with PVA to produce HTCC/PVA composite nanofiber membranes. The measured maximum filtration rates of PM10, PM2.5, and PM1.0 were about 92%, 86%, and 82% respectively, and the antibacterial rates of *Escherichia coli* and *Staphylococcus aureus* were both over 99% when the ratio of PVA-HTCC reached 6:4.

### 4.5. Discussion

The different and unique characteristics greatly explore the application range of nanofibrous membranes. In addition to the structures that have been discussed previously in this review, the usage and mixtures of materials are important factors that can greatly affect the characteristics of electrospun membranes. The spectrum of electrospinning materials includes conventional basic materials, green materials, and additional agents. With the addition of different agents, the membranes are given unique characteristics and functions without sacrificing the filtration performance. With the introduction of agents like PSA, ${\mathrm{BaTiO}}_{3}$, ZIF, etc., thermal stability is ensured. The antibacterial agents including ${\mathrm{AgTiO}}_{2}$, N-halamine, Ag_F made antibacterial function of electrospun membranes possible. In the future, the spectrum of electrospinning materials is surely expanding and awaits further exploration.

## 5. Applications in Air Filtration

As stated above, electrospun polymer membranes have already been proved highly effective against PM2.5 pollution. The unique features of high filtration efficiency and low pressure drop together guarantee the extensive application in the field of air filtration of PM2.5. The filtration performance is further enhanced by the development of electrospinning process, structures, and materials, making the electrospun membranes highly adaptable in individual protection both outdoor and indoor.

### 5.1. Outdoor Protection

The application of outdoor protection is concentrated on high-efficiency filtration masks. In addition to high filtration efficiency, low pressure drop is another key factor which is decisive to the actual usage of electrospun membranes, because as a piece of protective garment, masks should exhibit good air permeability and breathability. Moreover, these masks provide protection for individuals even in harsh conditions owing to their special advantages such as high porosity, high mechanical strength, and multi-functions like thermostability, antibacterial activity.

**Figure 12:**
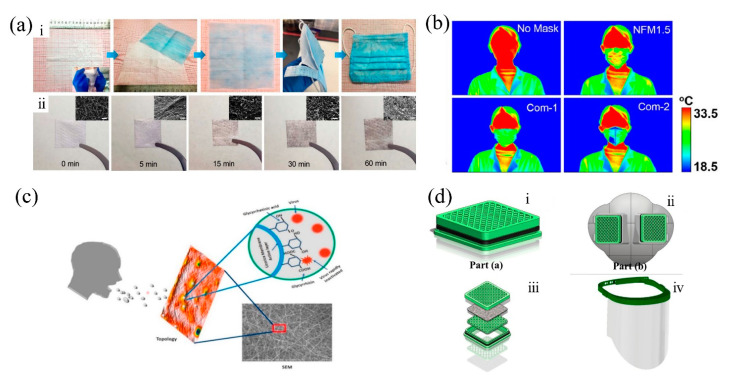
(**a**) (**i**). Photographs of the mask preparation process with 05GOPAN membrane as a wearable air filter. (**ii**) Photographs and SEM images (insets) showing the filter effect of 05GOPAN membrane after filtration of different times. Scale bars: 5 µm. Reprinted with permission from ref. [53]. Copyright 2018 Elsevier B.V. (**b**) Thermal images of bare face and faces covered with NMF1.5 and two commercial face masks. Adapted with permission from ref. [84]. Copyright 2018 Elsevier Inc. (**c**) Virus deactivation mechanism of the antiviral mask. Reprinted with permission from ref. [93]. Copyright 2020 Elsevier Inc. (**d**) Schematic representation of the design of the nanofibrous respirator face mask; (**i**) depicts the respirator filter containing multilayers of CuONPs/GO@PLA and CuONPs/GO@CA nanofibers. Part (**ii**) represents the fixed part of the face mask. The assembly of the multilayers consisting of nanofibers into a respirator filter is shown in (**ⅲ**). The face shield containing two parts and fabricated via the molding procedure is shown in (**ⅳ**). Reprinted with permission from ref. [92]. Copyright 2020 Elsevier Ltd.

As for the applications of PM2.5 filtration masks, Huang et al. [83] fabricated an efficient air filtration mat using electrospinning technique and solvent vapor annealing (SVA). During the SVA process, the fiber surface was wrinkled and therefore enhanced the PM2.5 filtration. Compared with commercial masks, this air filtration mat exhibited higher filtration efficiency under thick haze. Yang et al. [56] fabricated composite multilayer-structured membrane, which exhibited filtration efficiency of 99.3%, a low pressure drop of 64 Pa, and a good air permeability of 278 mm $s^{-1}$. The membrane was able to maintain the face cool and was comfortable even in a hygrothermal environment. Wang et al. [84] developed a fabrication strategy for electret nanofiber filter medium of PES/${\mathrm{BaTiO}}_{3}$ via electrospinning. The fabricated NFM1.5 mask displayed high filtration efficiency of 99.99%, low pressure drop of 67 Pa, and low basis weight of 4.32 ${g/m}^{2}$. More importantly, due to its ultralight basis weight and high porosity, NFM1.5 mask had a distinct radiative cooling effect, which guaranteed the wearing comfort while providing better protection than commercial face masks (Figure 12b). Šišková et al. [85] recycled ethylene terephthalate (PET) from domestic plastic waste and fabricated nanofibrous membrane via electrospinning. The results showed that the filter area and filter shape could improve the breathability of the membrane. Li et al. [53] fabricated masks using graphene oxide/polyacrylonitrile (GOPAN) composite membranes with an olive-like beads-on-a-string structure. The masks showed remarkable filtration efficiency of 99.97% for PM2.5 and low pressure drop of 8 Pa (Figure 12a). Huang et al. [86] fabricated nanofibrous protective masks from PAN and MC by electrospinning, and these masks displayed strong antimicrobial efficacies against both *S. aureus* and *E. coli* O157:HH7. Hashmi et al. [87] fabricated PAN/CuO nanofibers via electrospinning. The strength and the air permeability of the membranes were enhanced by the added copper oxide nanoparticles, and the prepared membranes showed good antimicrobial activity and release properties. Liu et al. [88] reported a new self-powered electrostatic adsorption mask based on the poly(vinylidene fluoride) electrospun membrane and a triboelectric nanogenerator driven by respiration. The mask showed that the removal efficiency of coarse and fine particles was higher than 99.2% and 86.9% for that of ultrafine particles.

To combat the new diseases caused by bacteria or viruses like coronavirus pandemic, researchers have been seeking solutions in nanotechnologies. Application of electrospun multifunctional nanofibrous filtration masks, serving as an effective physical protection, is a key strategy to prevent viral infection [89]. The most essential component of the filtration mask is the fibrous membrane used as the filtration layer [90], so the antivirus activity of the electrospun filtration membrane is the most decisive factor of the protection effect. Sivri et al. [91] introduced a novel face mask prototype. During the electrospinning process, vinyl alcohol and PVA/SAP were simultaneously coated onto face masks, resulting in their virus protection and comfort properties. The adsorbing capability was enhanced and the masks were able to absorb the sweat in the vapor form, and antivirus activity was reported. Ahmed et al. [92] reported a novel design from Egypt, utilizing a reusable, recyclable, customizable, antimicrobial, and antiviral mask by electrospinning (Figure 12d). To fabricate this newly designed antiviral mask, polylactic acid and cellulose acetate were combined with copper oxide nanoparticles. The polymeric network could block the airborne viral particles, and the added particles would further inactivate the bacteria and viruses. Chowdhury et al. [93] first fabricated an antiviral mask by electrospinning using antimicrobial licorice root extract mixed with PVA (Figure 12c). The results indicated that the fabricated mask’s diameter was about 15–30 μm with random porosity and orientation which have the capability to terminate the virus. The mask also exhibited good breathability, with porosity less than the size of COVID-19. He et al. [94] introduced a method of 3D printing based on electrospinning to make mask filters that were changeable in shape and biodegradable. PLA nanofiber web was fabricated, which had a self-reinforced hierarchical structure and a transparent look. The unique transparency of the mask had a positive effect on reducing communication barrier for wearers with mutism or hearing impairment, because lipreading is possible. In addition to electrospun masks, antimicrobial materials could be made into protective clothing, which had potential in providing extra and extensive protection. For example, Khanzada et al. [95] fabricated nanofiber membranes using aloe vera and polyvinyl alcohol (AV/PVA) via electrospinning, and these membranes showed excellent antimicrobial activity against *S. aureus* and *E. coli*. Xu et al. [96] developed a roll-to-roll method to fabricate masks based on fast transfer of electrospun nanofiber film from roughed metal foil to a receiving mesh substrate. This method, compared with direct electrospinning method, is ten time faster and the fabricated masks have better filtration performance and higher transmittance.

### 5.2. Indoor Protection

The indoor air quality, as one of the most important living conditions, is of residents’ concern because like outdoor PM pollution, the indoor air pollution is also a threat to public health because of the long time of exposure. Unlike the outdoor application of filtration masks, which are mostly worn outside, the indoor air filtration mainly relies on air filters to screen the PM off the household or other indoor settings. There are already several air filters in service [97], but the novel electrospun nanofiber membranes with exceptional filtration efficiency can easily outperform their commercial rivals.

**Figure 13:**
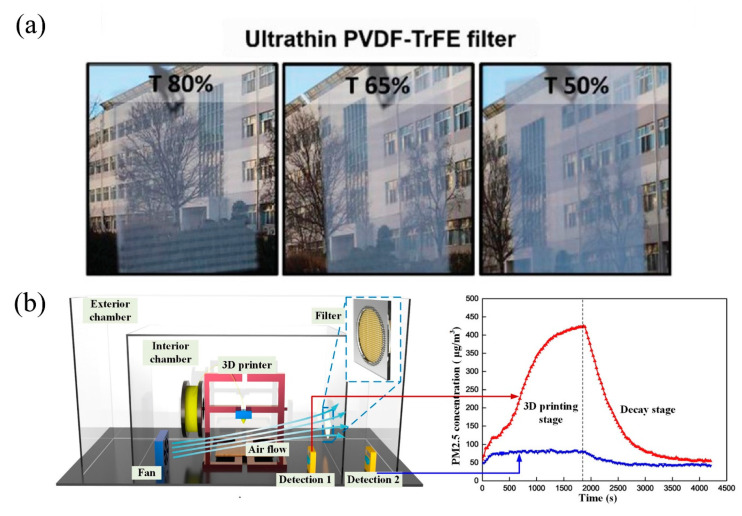
(**a**) Photographs of the PVDF-TrFE nanofiber filters with different light transmittances (T): 80%, 65%, and 50%. Adapted with permission from ref. [98]. Copyright 2019 Wiley-VCH Verlag GmbH & Co. KGaA, Weinheim. (**b**) Schematic of particles filtration experiment during 3D printing. Reprinted with permission from ref. [101]. Copyright 2019 Elsevier B.V.

As for the advanced electrospun filters, Han et al. [98] reported an electrically activated ultrathin PVDF-TrFE nanofiber air filter with high PM1.0 filtration efficiency of 94%. This nanofiber filter also exhibited good light transmittance of 65%, which allows it to be installed on the window frames of houses as an economically affordable way to prevent indoor air pollution instead of applying expensive air circulation systems (Figure 13a). Gobi et al. [99] produced composite electrospun membrane electret filter using PAN and ${\mathrm{SiO}}_{2}$NPs between nonwoven fabrics, and the fabricated membrane with ${\mathrm{SiO}}_{2}$ showed better filtration performance than commercial filter medium.

Another source of PM that possibly exists in the household is the industrial dust emitted from 3D printing, as 3D printers are becoming increasingly accessible. Rao et al. [100] studied the four different stages when PM was generated during the fused deposition modeling (FDM) 3D printing process, and fabricated PCL nanofiber-based air-filters to capture the emitted PM2.5 particles. Cao et al. [101] expanded the application scenarios of electrospun filters to industrial emissions like 3D printing. The electrospinning process was optimized using design of experiment (DOE), and bead-free PAN nanofibers with diameter of <100 nm was formed. The fabricated membranes were used to filter PM2.5 emissions from FDM 3D printing and the PAN membrane with diameter of 77 nm showed a filtration efficiency of 81.16% (Figure 13b).

### 5.3. Discussion

The most common applications of electrospun filtration membranes can be roughly concluded as outdoor protection and indoor protection, namely filtration masks and indoor air filters. As a wearable protection against air pollution, the application of filtration masks shows a trend toward good wearing comfort, extra functions that can offer protection even in harsh conditions, while the indoor air filters, which often serve as guards around the household, demand good transmittance, high durability, and low cost.

Despite the above eye-catching progress, there are still problems and challenges to be solved and tackled in the future for these electrospun membranes to become off-the-shelf productions. One is the disposal and recycle of the product after use. To solve this problem, the spectrum of electrospinning materials needs to be enlarged. Green and biodegradable materials introduced in Section 4.4 are ideal candidates for the environment-friendly disposal of these nanofibrous masks and filters. Furthermore, research on electrospinning using recycled materials needs to be done, as domestic plastic waste can be a promising source for the recycled polymers [85].

## 6. Summary and Future Perspectives

### 6.1. Summary

Air pollution caused by ambient PM2.5 is an environmental problem that cannot be ignored. Due to the advantages of high filtration efficiency, low pressure drop, high porosity, large specific area, good mechanical strength and multi-functions, nanofibrous membrane fabricated using electrospinning techniques have gained unshakeable status in the field of PM filtration. The development of novel electrospinning techniques, including needleless electrospinning, multi-needle electrospinning, and solvent-free electrospinning, is trending toward fast-speed, large-scale and non-toxic production. Unique structures such as bead-on-string and multilayer, along with different applications and combinations of electrospinning materials, gives membranes optimized filtration performance, characteristics and functions like thermal stability, antibacterial activity, and biodegradability. Finally, electrospun masks and filters are reliable and promising applications that can be used in both outdoor and indoor settings. It is concluded from the studies that the electrospun membranes as the PM filter medium has the following advantageous characteristics.

(1) Effective in protection against PM while maintaining good wearing comfort. Unique characteristics of electrospun membranes such as high porosity, low pressure drop, and different structures together allow air flow through the filter more easily, and the breathability and thermal comfort are therefore guaranteed, which are crucial factors of protective masks.
(2) Relatively easy to fabricate, and large-scale production is possible. The most widely used single nozzle electrospinning is simple to set up and operate. Other electrospinning techniques such as needleless and multi-needle electrospinning can simultaneously produce multiple polymer jets, supporting the large-scale and fast-speed production of membrane filters.
(3) Good versatility and adaptability to harsh conditions. With the introduction of different agents, various functions like thermal stability and antibacterial ability can be achieved. These diverse functions together with the notable filtration performance provide reliable protection even in harsh environments.

### 6.2. Future Perspectives

**Figure 14:**
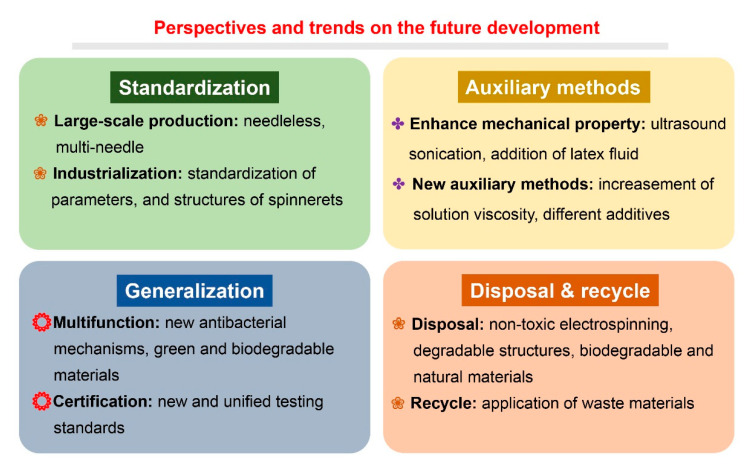
Perspectives and trends on the future development of electrospinning techniques and nanofibrous air filtration membranes.

Although electrospun nanofibrous filtration membrane and its application have already been studied deeply and extensively, there are still some possibilities and perspectives that need exploration, and more work is required in this context. Future perspectives and trends are summarized in Figure 14.

(1) Standardization and industrialization of novel electrospinning techniques. As the solid foundation of broad applications, new electrospinning techniques need to be standardized to guarantee mass production. The electrospinning techniques introduced in this review, especially multi-needle and needleless electrospinning, are suitable for large-scale production of nanofibrous membranes. However, only a small number of electrospinning apparatus have achieved large-scale and fast-speed production of nanofibrous membranes, like the electrospinning equipment mentioned in the work of Liang et al. [102] and the actual realization of these novel electrospinning techniques on the large-scale production and industrialization is still very limited and is a major challenge due to the control difficulty, unstable quality, lack of standardized testing procedures, etc. The diameter of electrospun membranes is affected by a number of parameters, like nozzle distance, voltage strength, solution flow rate, collection speed, ambient temperature, solution concentration, etc. Changes of these parameters will finally result in the deviation of filtration performance. In the industrialization of the novel electrospinning techniques, parameters of these techniques require standardization to guarantee the fast-speed and large-scale production of nanofibrous membranes with the expected filtration performance. Moreover, because multi-needle and needleless electrospinning techniques both include specially designed spinnerets with unique structures, structure of the highest production rate and the corresponding design parameters of this structure need to be determined and standardized.
(2) Auxiliary methods during the electrospinning process. Since the relatively weak mechanical property of membranes is a shared challenge faced by all types of electrospinning techniques, especially solution electrospinning, auxiliary methods that can enhance the mechanical property of nanofiber membranes are highly desirable. Ultrasound sonication prior to electrospinning process [103] and latex fluid as an additive to polymer solution [104] are two auxiliary methods that have been proved effective in the improvement of mechanical property. However, the related research is still rare. In the future, auxiliary mechanisms and methods, such as increasement of solution viscosity and introduction of different additives, need to be explored in both width and depth.
(3) Generalization of multifunctional masks and filters. Nanofibrous membranes have already proven to be promising in the realization of various characteristics such as thermal stability, antibacterial function, environment-friendliness, and biodegradability. In these unique characteristics, antibacterial function is most crucial to shield humans from hazardous bacteria and viruses, therefore the advanced antibacterial masks and filters need to be generalized and put into actual use. The research of antibacterial mechanisms such as new additional agents and surface functionalization, awaits further development. Another problem is the certification procedure of these filtration products. Before putting into the market, the filtration membranes need to be tested and evaluated to meet the standards. For example, in EU, air filters are certificated according to ISO 16890 and EN1822:2019, which require stringent filtration tests. However, the testing procedures of the newly produced membranes corresponding with the standards are still lacking. In future research of these novel filters, standardized tests should be done following the experiments. With the gradual industrialization process of novel nanofibrous membranes, the unified test standards of these products need to be set to evaluate the characteristics of membranes more precisely.
(4) Disposal and recycling of nanofibrous products. The inevitable problem of proper disposal and recycling of the electrospun air filtration membranes comes with the applications of these membranes. To meet the demand of environment-friendliness, research on the electrospun air filtration membranes is trending toward green, biodegradable, recyclable, and non-toxic materials. In the electrospinning techniques summarized in this review, the solvent-free electrospinning technique is bright in green and non-toxic production of nanofibrous membranes because it discards the toxic solvents. The structure of membranes should be designed as such to make it reusable and easily degradable. The spectrum of electrospinning materials needs to be expanded and reusable, green, and biodegradable materials deserve more focus.

## Figures and Tables

**Table 1:** Summary of needleless electrospinning.

Spinneret Design	Mechanisms	Refs.
Bubble	Under the effect of applied electric force and air pressure, polymer jets break the surface tension and are formed from bubbles on the liquid surface, then drawn toward the grounded collector.	[27,28]
Wire/coil	There are two ways of solution delivery: jets emerge from droplets formed on the wired spinneret through capillary effect, or splashed out by the rotating spinneret in the solution tank.	[29,30]
Disk of plate	Strong electric field is generated on the sharp edges of plate, disk or bowl, and therefore solution jets are formed there. Plates can be stacked into a waterfall composition.	[31,32]
Cylindrical	The cylindrical spinneret is either rotating or stationary. The rotating cylinder is first coated with solution, then generates jets under the applied electric field. The stationary cylinder is a vertically set rod, with solution provided by a syringe.	[33]
Tube with embedded wire loop	The jets are generated from the wire loop fixed on one end of the tube, with solution fed through the tube.	[25]
Sprocket wheel	The pivoting sprocket wheel is half immersed in solution tank. All the teeth dip in the solution and move to the top position, then the solution jets are formed on the edges of the teeth.	[34]

**Table 2:** Brief comparison of four electrospinning techniques.

	Single Needle	Needleless	Multi-Needle	Solvent-Free
**Pros**	Easy to set up and operate Easy maintenance Low cost	No clogging of spinneret Easy maintenance High production rate	Better controllability of the fiber distribution Easy to set up High production rate	No toxic solvents Low cost Direct writing of arranged nanofibers
**Cons**	Low production rate Clogging	Inconsistent solution concentration High voltage	Clogging Interaction of the jets	Thermal degradation of polymer Difficult to control pore size or fiber diameter
